# HBr or not HBr? That is the question: crystal structure of 6-hy­droxy-1,4-diazepane-1,4-diium dibromide redetermined

**DOI:** 10.1107/S2053229619005321

**Published:** 2019-05-16

**Authors:** Mateusz Piontek, Bernd Morgenstern, Nils Steinbrück, Bastian Oberhausen, Guido Kickelbick, Kaspar Hegetschweiler

**Affiliations:** a Inorganic Chemistry, Universität des Saarlandes, Campus C4.1, 66123 Saarbrücken, Germany; b Inorganic Solid State Chemistry, Universität des Saarlandes, Campus C4.1, 66123 Saarbrücken, Germany

**Keywords:** di­hydro­bromide, crystal structure, protonated di­amine, misinterpreted H atom, series-termination error

## Abstract

Liu *et al.* [*Chin. J. Struct. Chem*. (1996 ▸). **15**, 371–373] reported the structure of 6-hy­droxy-1,4-diazepane di(­hydrogen bromide), C_5_H_12_N_2_O·2HBr, which was inter­­preted in terms of neutral diazepane and HBr mol­ecules. We found, how­ever, ample evidence that the formation of an organic salt, consisting of a di­ammonium cation and two bromide anions, is more plausible. This inter­pretation is also in agreement with thermogravimetric analysis and with the observed solution behaviour. The crystal structure of 6-hy­droxy-1,4-diazepane-1,4-diium dibromide, C_5_H_14_N_2_O^2+^·2Br^−^, measured at 142 K, crystallized in the ortho­rhom­bic space group *P*2_1_2_1_2_1_. The structure displays O—H⋯Br and N—H⋯Br hydrogen bonding. Contact distances are given. A search in the Cambridge Structural Database for the singly-bonded H—Br moiety revealed a total of 69 structures. The question, whether these structures really include HBr as neutral mol­ecules or rather Br^−^ anions and a protonated substrate such as an amine, is addressed.

## Abstract 2

A probably wrong inter­pretation of a previously determined structure of the title compound is discussed and an alternative more convincing description is presented.

## Introduction   

6-Hy­droxy-1,4-diazepane (dazol) was first synthesized by Saari *et al.* (1971 ▸) and has been used as a tridentate facially coordinating metal-complexing agent (Liu *et al.*, 1997*a* ▸). The free ligand has been isolated as a di­hydrogen bromide, C_5_H_12_N_2_O·2HBr, and its crystal structure has been reported [Liu *et al.*, 1996 ▸; Cambridge Structural Database (CSD; Groom *et al.*, 2016 ▸) refcode TOKTIW]. The authors postulated crystallization of the neutral diazepane as a free base together with two HBr mol­ecules. From a chemical point of view, the formation of discrete HBr mol­ecules beside a basic entity is surprising, even taking into account that the situation in the solid state does not necessarily reflect the well-known acid–base properties in aqueous solution. However, a search for mol­ecular H—Br in the CSD (Version 5.20, 2018) gave a total of 69 hits and hence some support for the mol­ecular 
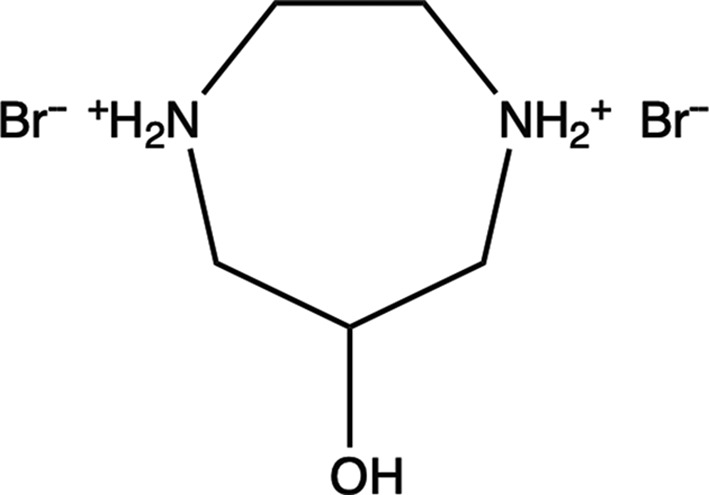
model. To shed light on this discrepancy, we have: (i) investigated the chemistry of dazol in aqueous solution using potentiometric titration experiments and pD-dependent ^1^H NMR spectroscopy, and (ii) prepared a crystalline sample of the title compound (see Scheme[Chem scheme1]), repeated the structure determination reported by Liu *et al.* (1996 ▸) and performed additional thermogravimetric measurements to elucidate the solid-state properties.

## Experimental   

### Synthesis and crystallization   

The synthesis of the title compound was performed following the protocol given by Saari *et al.* (1971 ▸) with minor modifications. *N*,*N*′-Dazolbis(toluene­sulfonamide) was pre­pared as described by the reaction of *N*,*N*′-ethyl­ene­bis(toluene­sul­fon­amide) with 2,3-di­bromo­propan-1-ol. How­ever, in the next step, the detour *via* the ­acetate proved not necessary. The two toluene­sulfonamide groups could be removed directly without any loss of yield by heating the bis­(toluene­sulfonamide) sus­pended in 48% aqueous HBr to 125 °C for 3 h. The clear bright-yellow solution was allowed to cool to room temperature and was then evaporated to dryness under reduced pressure. The resulting solid was washed with diethyl ether and ethanol to yield the title compound as a pale-gray solid (91%). Crystals were grown by slow diffusion of EtOH into an aqueous solution of the product which has been acidified with additional HBr.

### Refinement   

Liu *et al.* (1996 ▸) reported an unambiguous location of the C-, N-, O- and Br-atom positions of one dazol moiety and two crystallographically independent Br atoms. It is clear that a reliable assignment of H-atom positions is more difficult, and might even be a highly questionable task, if the high electron density of the two heavy Br atoms is considered. Unfortunately, the data set of Liu *et al.* (1996 ▸) is of rather poor quality. According to the CSD, the data set was recorded at room temperature. Moreover, the information provided by these authors is not really conclusive. In their Table 1 ▸ (and in the CIF available from the CSD), the H-atom positions are all listed without standard deviations and the authors stated that ‘H atoms were located by geometric method except the hydroxyl one, which was oriented from difference Fourier map.’ This statement seemingly indicates that the H(—Br), H(—C) and H(—N) positions have not been taken from a difference Fourier map. It is confusing that some of the C—H (1.13 Å) and N—H (1.15 Å) distances and H—C—H (93.2°) angles do not fall in expected ranges. Obviously, the authors did not apply the usual riding model with fixed angles and distances. The two H—Br lengths of 1.04 and 1.08 Å are also rather short.

To improve the quality of the data set, we performed a data collection at −131 °C. The space group and lattice parameters, as well as the positional parameters of the non-H atoms, were all in agreement with Liu’s report (Liu *et al.*, 1996 ▸). Crystal data, data collection and structure refinement details are summarized in Table 1 ▸. Inspection of a difference Fourier map unambiguously yielded all of the H(—C) and H(—O) hydrogens with meaningful bond lengths and angles. The positional parameters of these H atoms were now included in a subsequent refinement using a riding model, with an appropriate restraint for H(—O) and appropriate constraints for the H(—C) atoms. At this stage, the refinement provided agreement factors of *R*_1_ = 3.94% and *wR*_2_ = 7.47%. The two most intense peaks, with electron densities of 0.72 and 0.70 e Å^−3^, in the new difference Fourier map were located in proximity to atoms N2 and N1, respectively. They were inter­preted as H(—N) positions. Further refinement con­firmed this assignment and gave a slight drop of the *R*_1_ (3.85%) and *wR*_2_ (6.64%) values. A subsequent difference Fourier map exhibited more than 30 unassigned peaks with electron densities in the range 0.62–0.42 e Å^−3^. The four peaks with highest intensities (*Q*1–*Q*4) were located in proximity to atoms N1, Br2, N2 and Br1 (in this order). At this stage, two different models were considered for the final refinement. Model *A* comprised *Q*1 and *Q*3, which were both inter­preted as H(—N) positions; *Q*2 and *Q*4 were disregarded. Model *B* comprised *Q*2 and *Q*4 as H(—Br) positions, with *Q*1 and *Q*3 now being neglected. Free refinement of model *A* resulted in a stable and meaningful result, yielding two NH_2_^+^ groups, whereas the free refinement of model *B* collapsed: the H(—Br) atoms moved to positions with very short Br—H distances (<0.3 Å). The agreement factors of model *A* (*R*_1_ = 3.72% and *wR*_2_ = 6.31%) were marginally better than those of model *B* (*R*_1_ = 3.84% and *wR*_2_ = 6.63%). These results clearly show that the crystal structure analysis alone does not allow discrimination with certainty between the two models. However, the stable refinement of model *A* and the slightly better agreement factors may be regarded as a first sign for the ionic structure. The observed electron density in proximity to the Br atoms (*Q*2 and *Q*4) could be understood as well-known series-termination errors in the Fourier synthesis (Glusker *et al.*, 1994 ▸).

In the final refinement (model *A*), a riding model was used for the C-bonded H atoms. As suggested by Müller *et al.* (2006 ▸), the positional parameters of the O- and N-bonded H atoms were refined using isotropic displacement parameters, which were set at 1.5*U*_eq_(O) or 1.2*U*_eq_(N) of the pivot atom. In addition, restraints of 0.84 and 0.88 Å were used for the O—H and N—H distances, respectively.

## Results and discussion   

### Chemical context   

**Figure 1:**
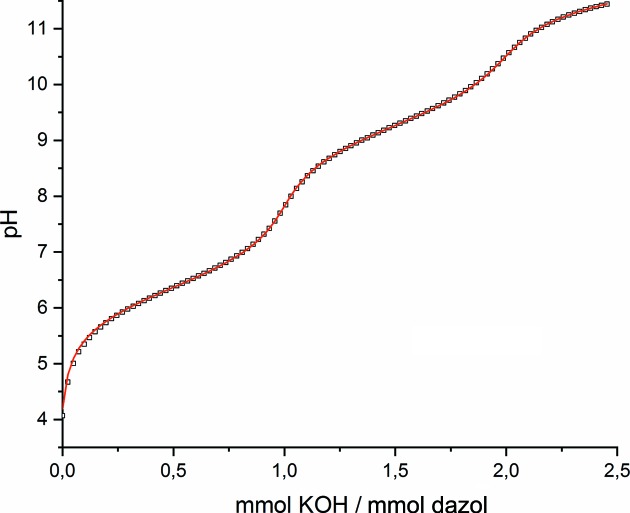
Titration curve (25 °C, 0.1 *M* KCl) of H_2_dazol^2+^. Squares refer to the measured values. The red line was calculated using the deprotonation constants −log *K*_a,1_ = 6.01 and −log *K*_a,2_ = 9.05.

**Figure 2:**
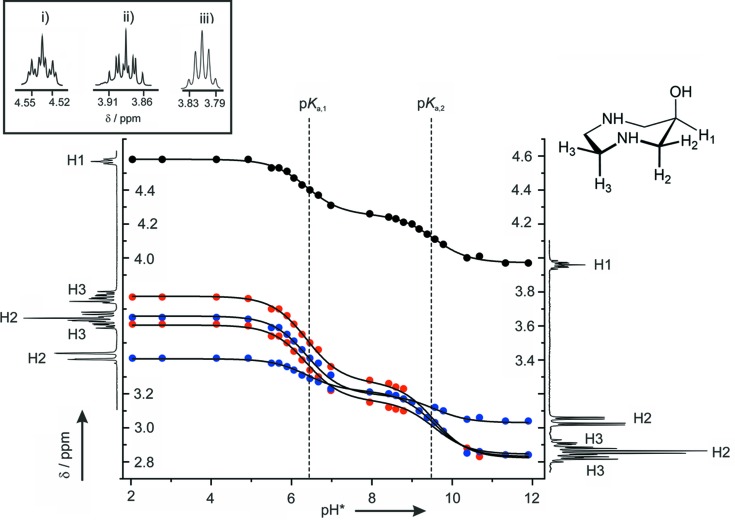
pH* dependence of the various ^1^H resonances for the nonlabile protons (pH* refers to the uncorrected pH-meter reading in D_2_O for an electrode calibrated in H_2_O). The observed resonances (δ_obs_) are shown as closed circles: H1 (black), H2 (blue) and H3 (red). The lines were calculated [minimization of Σ(δ_obs_ − δ_calc_)^2^]. Inset: The pattern of H1 at (i) pH* = 5, (ii) pH* = 10 and (iii) in 5 mol l^−1^ NaOD.

Liu *et al.* (1996 ▸) postulated the presence of neutral diazepane as a free base, together with two mol­ecular HBr units in the solid-state structure. At first glance, such an inter­pretation is amazing, since HBr is known to react as a very strong acid, and the diazepane moiety – as an alicyclic di­amine – is expected to react as a base. We investigated the protonation behaviour of dazol in aqueous solution (25 °C) and found – as expected – that an uptake of two protons occurred readily upon addition of acid. A series of potentiometric titration experiments (Fig. 1 ▸) revealed two p*K*_a_ values of 6.01 and 9.05 (0.1 *M* KCl) or 6.37 and 9.28 (1 *M* KNO_3_) for H_2_dazol^2+^. These values are in agreement with those reported for related amino alcohols (Martincigh & Marsicano, 1995 ▸). In addition, we also performed a ^1^H NMR titration experiment in D_2_O (Fig. 2 ▸) and observed characteristic pD-dependent resonances for the H(—C) protons upon addition of NaOD. This pD dependency could again be inter­preted as a twofold deprotonation reaction of the dication. The evaluated p*K*_a_ values in this medium are 6.35 and 9.62. All these characteristics clearly indicate that addition of two equivalents of HBr to an aqueous solution of dazol results in a complete transformation into the H_2_dazol^2+^ dication. Crystal growth of the title compound has indeed been performed in such an acidic aqueous medium. However, one must of course be aware that – in general – the solid state does not necessarily depict the equilibrium composition in solution.

**Figure 3:**
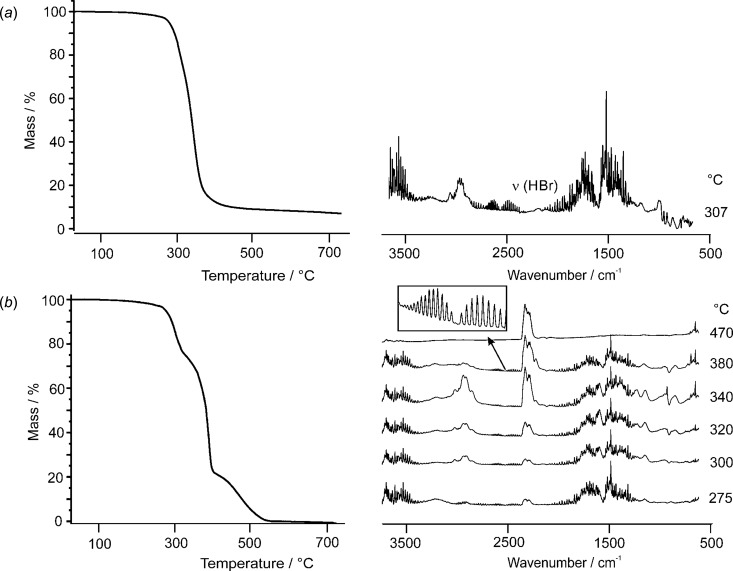
Thermogravimetric analyses of H_2_dazolBr_2_ (%weight *versus T*) together with IR characteristics of the evolving gases at temperatures as indicated. Heating of the sample (*a*) in a steady stream of N_2_ only and (*b*) in a 1:1 mixture of N_2_ and O_2_. The inset in (*b*) shows an enlargement for the range 2400–2700 cm^−1^, indicating the formation of HBr in small traces.

A solid sample of the title compound was therefore investigated by IR spectroscopy, looking at around 2600 cm^−1^ for any H—Br stretch vibration. However, these measurements were not conclusive, since a possible H—Br peak was covered by the intense and broad absorption between 2200 and 3500 cm^−1^, caused by the various associated N—H and O—H stretching vibrations. Thermogravimetric measurements com­bined with an IR analysis of the gaseous products was more instructive (Fig. 3 ▸). A 20 mg sample was heated by a rate of 10 °C min^−1^ from room temperature up to 800 °C and exposed to a steady stream of N_2_ (20 ml min^−1^). Complete degradation occurred almost qu­anti­tatively (>90%) in one single step in the range 300–400 °C. Evolution of HBr could readily be recognized in the IR spectrum (2400–2700 cm^−1^) by its characteristic pattern for the two isotopomers with resolved transitions for the various rotamers (NIST, 2019 ▸). In addition, an organic component (O—H, N—H and C—H, and possibly C—C and C—O stretching vibrations, but no CO_2_) was formed. These findings are in agreement with a predominant sublimation of the product. It is well known that NH_4_Br and its organic derivatives, such as methyl­ammonium bromide sublimate in the range 300–400 °C (Ivanov *et al.*, 2019 ▸). The remaining small nonvolatile residue (5–10%) probably indicates some minor decomposition during the sublimation process (formation of elemental carbon, as indicated by a black coating inside the crucible). If a 1:1 stream of N_2_ and O_2_ (each 20 ml min^−1^) is applied to the sample during heating, the degradation occurred in more than one step. Again, less than 1% weight loss was noted below 200 °C. Up to 250 °C, the sample weight decreased slightly by about 3%. A first significant step of decomposition was then observed in the range of about 250–330 °C, with a corresponding weight drop of 21%. This value is clearly smaller than the 29% required for a dissociation of one HBr mol­ecule. The final part of the decomposition reaction occurred in two steps at 350–400 (−50%) and 400–550 °C (−22%). The IR spectra of the evolving gases showed the formation of H_2_O during the entire decomposition reaction. At elevated temperatures (>300 °C), increasing formation of CO_2_ and of some organic components (observation of C—H stretching vibrations) was also noted. Above 450 °C, CO_2_ remained as the most significant decomposition product. Inspection of the spectra did again exhibit that small traces of HBr have been formed. Maximum HBr production was found around 350–400 °C. These observations do not indicate a simple and qu­anti­tative loss of HBr at low temperature. It rather appears that small amounts of HBr are formed *in situ* during the entire decomposition process, particularly at elevated temperatures. As a conclusion, evolution of HBr appears generally to be combined with a complete breakdown of the entire structure.

### Structural commentary   

Eliel *et al.* (1965 ▸) proposed high conformational flexibility for cyclo­heptane, with a twist–chair (TC) conformation being of lowest energy. We previously studied 6-amino-1,4-diazepane (daza) as a metal-complexing agent, and the adoption of such a TC conformation for the seven-membered 1,4-di­aze­pane ring of H_3_daza^3+^ could indeed be confirmed by crystal structure analysis (Romba *et al.*, 2006 ▸; Neis *et al.*, 2010 ▸). Its ^1^H NMR spectrum exhibited a total of five resonances for the H(—C) protons, indicating a rapid inter­conversion of different conformations, yielding an averaged structure of higher symmetry (Longuet-Higgins, 2002 ▸). The mol­ecular structure of daza and dazol is closely related and similar ^1^H NMR characteristics have also been observed for dazol. The coupling pattern of the H—(C—*X*) proton (H_3_daza^3+^: *X* = NH_3_^+^; H_2_dazol^2+^: *X* = OH) revealed, however, some characteristic differences for the two compounds. For H_3_daza^3+^, this signal appeared as a triplet of triplets with one large coupling constant of 10.5 Hz and a second much smaller constant of 3.1 Hz. The large coupling of 10.5 Hz is indicative of a staggered orientation of the H—C—C(NH_3_^+^)—H fragment, with a torsion angle close to 180°. Obviously, the primary ammonium group of H_3_daza^3+^ is placed in an equatorial position. How­ever, for H_*x*_dazol^*x*+^ (*x* = 0, 1, 2), the corresponding coupling constants are significantly smaller, with a value of 5.4/1.4 Hz at pD 5.5 and 5.8/4.4 Hz at pD 10 (Fig. 2 ▸). It thus appears that in solution the hy­droxy group of H_*x*_dazol^*x*+^ (*x* = 0, 1, 2) is positioned axially. Inter­estingly, at a very high base concentration (5 mol l^−1^ NaOD), the signal of this proton is again shifted by about 0.2 ppm to lower frequency and appeared as a quintet with one unique coupling constant of 4.0 Hz. Obviously, the hy­droxy group of dazol becomes deprotonated in such a highly alkaline medium.

In agreement with our NMR study, the H_2_dazol^2+^ cation also adopted a TC conformation in the crystal structure, with the pivot atom N1 located in the isoclinal position. The puckering parameters (Boessenkool & Boeyens, 1980 ▸) of the seven-membered diazepane ring are *Q* = 0.821 (7) Å, *Q*2 = 0.506 (7), *Q*3 = 0.647 (7), φ_2_ = 86.5 (8)° and φ_3_ = 92.0 (6)°. Also, in accordance with the solution structure, the hy­droxy group adopted an axial position. The different orientation of the NH_3_^+^ group of H_3_daza^3+^ and the OH group of H_*x*_dazol^*x*+^ (*x* = 0, 1, 2) is remarkable. Since this difference is observed in the solid state, as well as in solution, the peculiar structure may be explained by the well-known attractive *gauche* effect (Entrena *et al.*, 1997 ▸), which proposes the preferential adoption of a *gauche* rather than a *trans* conformation for such an *X*—C—C—O*R* fragment (*X* = O, N).

**Figure 4:**
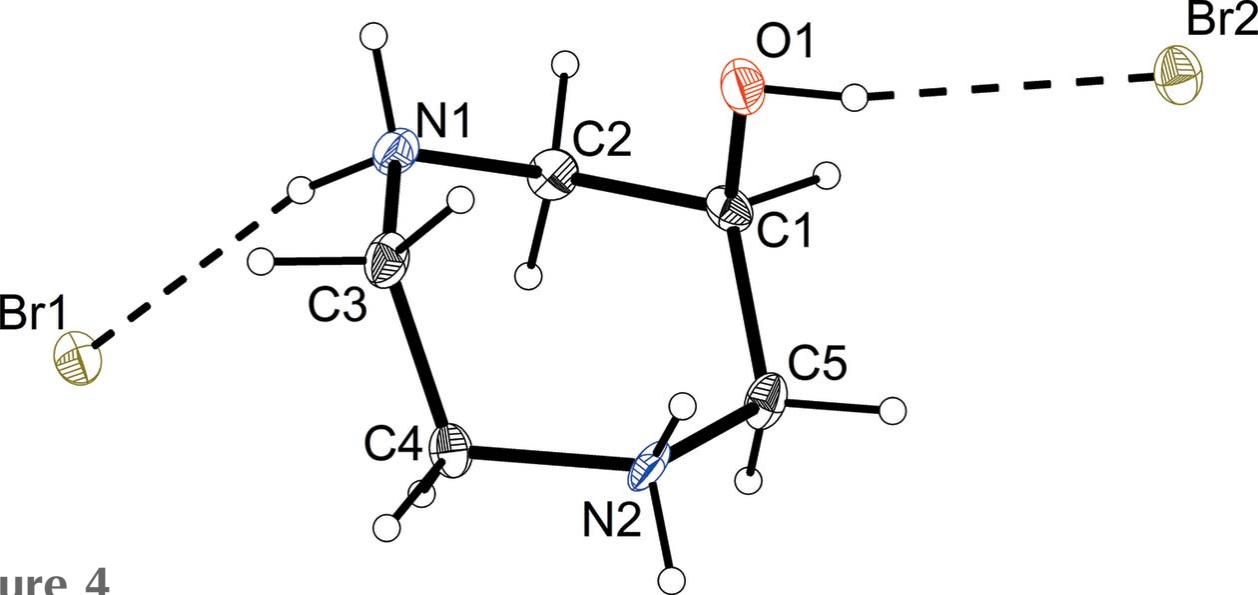
The mol­ecular structure of the H_2_dazol^2+^·2Br^−^ unit, with the atom-numbering scheme and displacement ellipsoids drawn at the 50% probability level.

Similar to the work performed by Liu *et al.* (1996 ▸), the crystal structure analysis presented here exhibited low precision, *i.e.* large standard uncertainties for bond angles and dis­tances. An inspection of the displacement parameters of the C, N and O atoms indicated some significant deviations for the displacement ellipsoids from a spherical shape (Fig. 4 ▸). Considering the high conformational flexibility of the seven-membered diazepane frame, we attribute these large deviations to minor disorder rather than to thermal motion (in contrast to Liu’s work, we performed data collection at −131 °C). It was, however, not possible to resolve this slight disorder in terms of a superposition of distinct individual conformers.

### Supra­molecular features   

**Figure 5:**
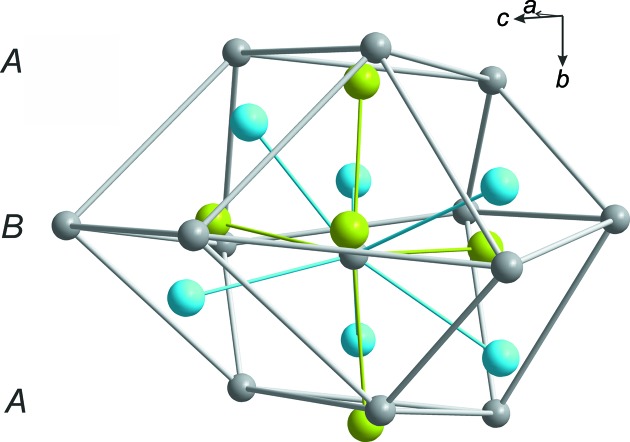
The distorted anti­cubocta­hedron formed by the gravicentres of 12 H_2_dazol^2+^ cations (gray spheres) together with the central H_2_dazol^2+^ cation. The two crystallographically independent Br^−^ anions are placed in 50% of the trigonal holes of a hexa­gonal layer (Br1 = green spheres) and in all of the octa­hedral holes (Br2 = blue spheres).

**Figure 6:**
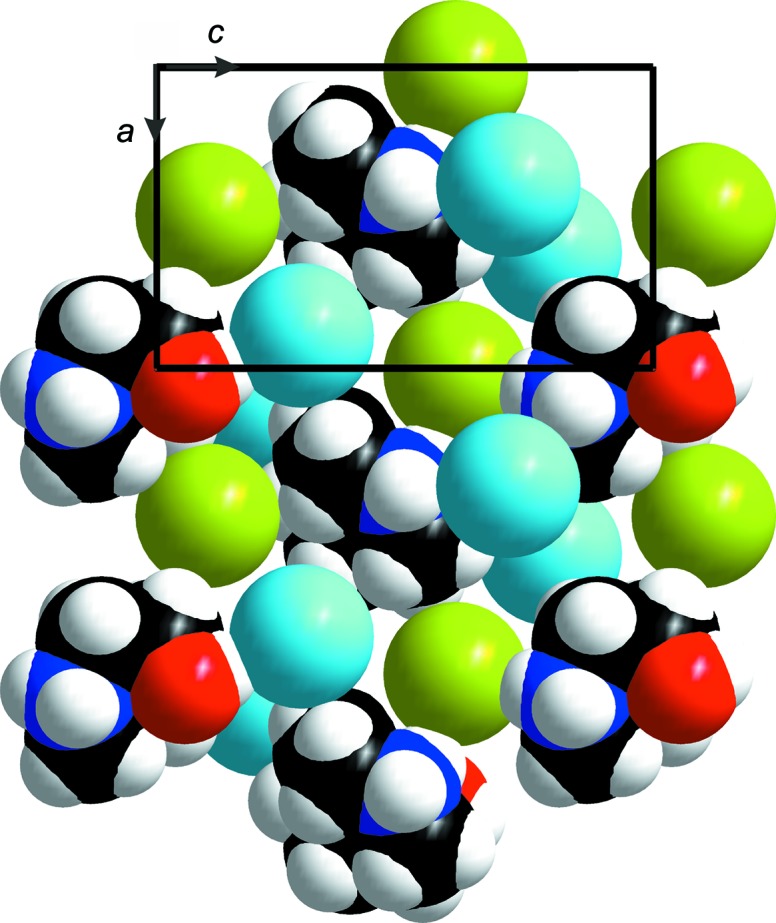
View of the (010) plane (space-filling model). The H_2_dazol^2+^ cations form a pseudohexa­gonal layer with the Br1 anions (green spheres) located in every second trigonal hole of this layer. The Br2 anions are shown as light-blue spheres and are placed in the octa­hedral holes above and below this layer. C, H, N and O atoms are shown as black, light-gray, dark-blue and red spheres, respectively.

**Figure 7:**
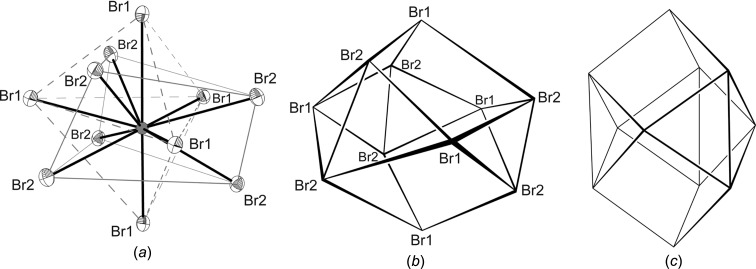
(*a*) The H_2_dazol^2+^ cation is surrounded by 11 Br^−^ anions. A coordination number (CN) of 11 is generated by the six Br2 anions forming a trigonal prism (tp, thin solid lines) and the five Br1 anions forming a trigonal bipyramid (tbipy, broken lines). The five vertices of the tbipy are placed over the mid-points of the five planes of the tp. (*b*) The observed distorted and (*c*) the idealized undistorted *D*_3*h*_ model (Edshammar polyhedron).

**Figure 8:**
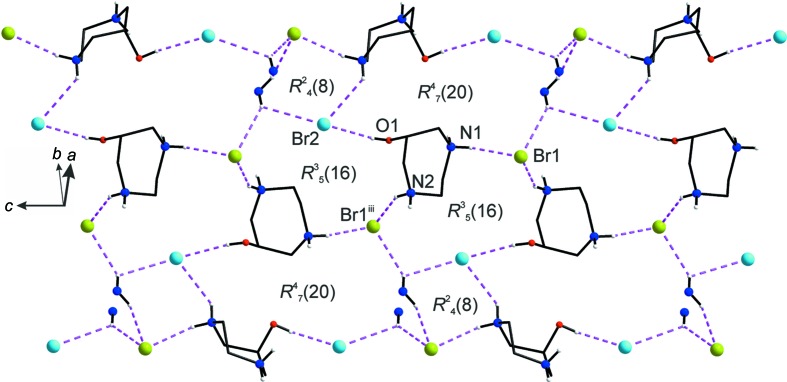
The hydrogen-bonding network observed for H(—N) and H(—O) H atoms as donors, and Br1 (green) and Br2 (blue) as acceptors. The plane perpendicular to the [1
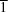
0] direction is shown. The resulting cyclic structures are characterized by the descriptors 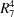
(20), 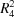
(8) and 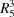
(16). [Symmetry code: (iii) −*x* + 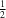, −*y* + 1, *z* + 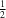.]

The cationic H_2_dazol^2+^ gravicentres (dgcs) are arranged into layers oriented parallel to the crystallographic *ac* plane. In these layers, each dgc is surrounded by six neighbouring dgcs, forming a distorted hexa­gon. The layers are stacked along *b* in a staggered fashion (*ABABAB*…) and can thus be regarded as a distorted hexa­gonal packing. If the two adjacent layers are taken into account, each dgc receives 12 dgc neighbours, which form an anti­cubocta­hedron (Fig. 5 ▸). However, some characteristic deviations from a regular shape are noted for this polyhedron. One reason for the distortion originates from the significant deviation of the H_2_dazol^2+^ cations from a spherical shape; these cations should be regarded as disks rather than spheres. Consequently, the (dgc)_12_ anti­cubocta­hedron is compressed along the pseudohexa­gonal axis, as is expressed by the unequal edge lengths (5.59–7.87 Å) and short inter­layer distances. Further distortion originates from the general position of the dgc and the reduced crystallographic symmetry. As a consequence, the dgc layers (and thus the equators of the anti­cubocta­hedra) are puckered. The symmetry class of this layer group is *p*12_1_1 (Inter­national Tables for Crystallography, 2002 ▸; Shubnikov & Koptsik, 1974 ▸). Inter­estingly, the Br1 ions (green spheres in Figs. 5 ▸ and 6 ▸) are located neither in the tetra­hedral nor in the octa­hedral holes of this packing. They are placed almost straight within the pseudohexa­gonal dgc planes. Each Br1 anion is thus surrounded by three dazol dications. Only 50% of these triangular holes are occupied. Such a packing becomes understandable if the huge difference in size between Br^−^ and H_2_dazol^2+^ is considered (Fig. 6 ▸). The entire geometry and coordination number of Br1 becomes evident if the staggered arrangement of the dgc layers is taken into account. Beside the three dgc neighbours of the triangular hole, the Br1 anions receive two additional dgc neighbours from adjacent dgc layers and the coordination polyhedron of Br1 can thus be described as a trigonal bipyramid. The Br2 ions (blue spheres) are located in the octa­hedral holes of the pseudohexa­gonal dgc packing. The coordination number of Br2 is thus six and the coordination geometry is a distorted octa­hedron. The H_2_dazol^2+^ cations, in turn, are surrounded by 11 Br anions with Br⋯dgc distances ranging from 4.0 to 6.5 Å (Fig. 7 ▸ *a*). The resulting Br_11_ structure can be described as a distorted Edshammar polyhedron (Fig. 7 ▸ *b*; Edshammar, 1969 ▸). Notably, the regular Edshammar polyhedron adopts *D*_3*h*_ symmetry (Fig. 7 ▸ *c*) and is a space filler (Lidin *et al.*, 1992 ▸). Each H_2_dazol^2+^ entity forms N—H⋯Br and O—H⋯Br hydrogen bonds (Table 2 ▸). Br1 is bonded to H1N—N1, H3N—N2(−*x* + 1, *y* + 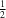, −*z* + 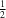) and H4N—N2(−*x* + 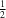, −*y* + 1, *z* − 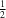). Further Br1⋯H inter­actions (Br1⋯H2N—N1, Br1⋯H1—C1, Br1—H2*A*–C2 and Br1⋯H3*B*—C3), with Br⋯H—N or Br⋯H—C angles of 131–140° and Br⋯H distances of 2.95–3.05 Å, must be regarded as very weak hydrogen bonding if they are to be regarded as hydrogen bonding at all. The sum of the van der Waals radii of Br and H is 2.95 Å (Bondi, 1964 ▸). Br2 forms only two unambiguous hydrogen bonds to H1O—O1 and H2N—N1(*x* + 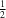, −*y* + 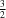, −*z* + 1). Again, the contacts Br2⋯H3*A*—C3, Br2⋯H4*A*—C4, Br2⋯H3N—N2, Br2⋯H2N—N1, Br2⋯H2*B*—C2, Br2⋯H5*A*—C5, Br2⋯H3*A*—C3, with Br⋯H distances of 2.88–3.05 Å and Br⋯H—N or Br⋯H—C angles of 117–162° may be considered as further weak inter­actions which stabilize the structure. A graph-set analysis (Bernstein *et al.*, 1995 ▸) shows the hydrogen-bonding network in the [1
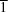
0] direction (Fig. 8 ▸). The Br1 and Br2 anions are μ_3_-acceptors and the N—H and O—H hydrogens are donors in three distinct ring systems, *i.e.* 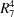
(20), 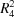
(8) and 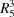
(16). Notably, there is no direct H_2_dazol^2+^⋯H_2_dazol^2+^ hydrogen bonding and, consequently, the hy­droxy group of H_2_dazol^2+^ does not act as an acceptor. All these structural features correspond well to the packing of large *charged* mol­ecular entities, as observed for instance in Zintl phases (Lidin *et al.*, 1992 ▸), and thus provide support for the ionic model.

### Database survey   

By studying some related literature, we became aware that the report of Liu *et al.* (1996 ▸) might not be the only example where the nature of ‘HBr’ in a crystal structure should be questioned. A search in Version 5.20 (2018) of the Cambridge Structural Database (CSD; Groom *et al.*, 2016 ▸), looking for mol­ecular H—Br (*i.e.* for an H atom directly connected to a Br atom by a single bond), revealed a total of 69 entries (see supporting information for the full list). Some of the listed entries do not provide three-dimensional coordinates and are thus not relevant for this discussion. Furthermore, it appears that the technical term ‘hydro­bromide’ may have led to confusion, since it has been used by some of the authors for a salt of a protonated organic mol­ecule and bromide as counter-ion, but appeared in our search as mol­ecular H—Br. For some of the listed compounds, the presence of HBr mol­ecules in the structure might be quite feasible from a chemical point of view. However, there remained still a total of 13 entries [CSD refcodes BEPQIY (Růžička *et al.*, 2013 ▸), EVAMIX (Cocco *et al.*, 2004 ▸), GICSOC (Aureggi *et al.*, 2013 ▸), KEKQAS (Wang *et al.*, 1999 ▸), KONVEO (Lin *et al.*, 1990 ▸), MOMVIV (Surendra Dilip & Gowri, 2014 ▸), MOMVIV01 (Gowri *et al.*, 2015 ▸), MUFKON (Liang *et al.*, 2002 ▸), NAVFOI01 (Zhao *et al.*, 2017 ▸), NIJGOC (Liu *et al.*, 1997*b* ▸), SOCZUH (Banothu *et al.*, 2014 ▸), UNESAI (Zhang & Shen, 2011 ▸) and YOTSIJ (Monte *et al.*, 1995 ▸)], where the simultaneous presence of HBr with a basic moiety (in KEKQAS, HBr and OH^−^!) is proposed, with H—Br bond lengths ranging from 0.79 to 1.84 Å. It is clearly beyond the scope of this contribution to decide whether these structural assignments are correct. However, it appears that: (i) the maintainers of the database should carefully clarify whether the expression ‘hydro­bromide’ refers to an ionic or rather a mol­ecular model, and (ii) reporting such a crystal structure, authors should take the required care to clarify the bonding mode when postulating incorporation of undissociated HBr together with a basic moiety. Similar considerations might also be applicable for the so called ‘hydro­chlorides’ (292 entries for a H—Cl mol­ecule) and ‘hydro­iodides’ (20 entries for a H—I mol­ecule).

## Conclusion   

The solution and solid-state properties of the title compound do not provide evidence for a simple incorporation of HBr as intact mol­ecules into the solid-state structure. An ionic model, comprising one H_2_dazol^2+^ cation and two Br^−^ anions is clearly a better explanation. According to the structure postulated by Liu *et al.* (1996 ▸), the two HBr mol­ecules would not be bonded to other moieties by strong inter­actions (such as Coulombic forces or hydrogen bonding). The (Br1—)H⋯H(—Br2) separation of 2.24 Å roughly corresponds to the sum of the van der Waals radii of two H atoms (2.20 Å; Bondi, 1964 ▸) and, as a consequence, the two HBr mol­ecules would not act as H-atom donors in hydrogen bonds. One could thus expect that liberation of HBr should occur readily even at moderate temperatures.

For further clarification of this question, we grew single crystals of the title compound and repeated the X-ray analysis. We have shown that it is possible to refine the crystal structure in terms of an ordinary ammonium salt (see §2.2, *Refinement*). There is thus no need to postulate the rather exotic incorporation of mol­ecular HBr into the crystal structure. We think that, in such a case, it is more advisable to chose the model which also directly explains the observed chemical properties (acid–base behaviour and breakdown of the structure upon HBr elimination above 300 °C).

## Supplementary Material

Crystal structure: contains datablock(s) I. DOI: 10.1107/S2053229619005321/ku3242sup1.cif

Structure factors: contains datablock(s) I. DOI: 10.1107/S2053229619005321/ku3242Isup2.hkl

Details of the CSD search. DOI: 10.1107/S2053229619005321/ku3242sup3.pdf

CCDC reference: 1910784

## Figures and Tables

**Table 1:** Experimental details

Crystal data	
Chemical formula	C_5_H_14_N_2_O^2+^·2Br^−^
*M*_r_	278.00
Crystal system, space group	Orthorhombic, *P*2_1_2_1_2_1_
Temperature (K/°C)	142/−131
*a*, *b*, *c* (Å)	7.7005 (4), 9.2774 (5), 12.6853 (6)
*V* (Å^3^)	906.25 (8)
*Z*	4
Radiation type	Mo *K*α
μ (mm^−1^)	8.89
Crystal size (mm)	0.23 × 0.07 × 0.02

Data collection	
Diffractometer	Bruker APEXII CCD
Absorption correction	Multi-scan (*SADABS*; Krause *et al.*, 2015 ▸)
*T*_min_, *T*_max_	0.576, 0.746
No. of measured, independent and observed [*I* > 2σ(*I*)] reflections	4858, 2089, 1789
*R*_int_	0.050
(sin θ/λ)_max_ (Å^−1^)	0.652

Refinement	
*R*[*F* ^2^ > 2σ(*F* ^2^)], *wR*(*F* ^2^), *S*	0.037, 0.063, 0.93
No. of reflections	2089
No. of parameters	106
No. of restraints	5
H-atom treatment	H atoms treated by a mixture of independent and constrained refinement
Δρ_max_, Δρ_min_ (e Å^−3^)	0.61, −0.51
Absolute structure	Flack *x* determined using 646 quotients [(*I* ^+^) − (*I* ^−^)]/[(*I* ^+^) + (*I* ^−^)] (Parsons *et al.*, 2013 ▸)
Absolute structure parameter	0.05 (2)

Computer programs: *APEX2* (Bruker, 2010 ▸), *SAINT* (Bruker, 2010 ▸), *SHELXT2014* (Sheldrick, 2015*a* ▸), *SHELXL2014* (Sheldrick, 2015*b* ▸), *DIAMOND* (Brandenburg, 2007 ▸) and *PLATON* (Spek, 2009 ▸).

**Table 2:** Hydrogen-bond geometry (Å, °)

*D*—H⋯*A*	*D*—H	H⋯*A*	*D*⋯*A*	*D*—H⋯*A*
O1—H1O⋯Br2	0.84 (1)	2.41 (2)	3.244 (4)	170 (8)
N1—H1N⋯Br1	0.88 (1)	2.36 (2)	3.230 (6)	167 (7)
N1—H2N⋯Br2^i^	0.88 (1)	2.79 (6)	3.323 (6)	120 (5)
N2—H3N⋯Br1^ii^	0.88 (1)	2.69 (5)	3.422 (6)	142 (6)
N2—H4N⋯Br1^iii^	0.88 (1)	2.55 (3)	3.355 (6)	153 (6)

Symmetry codes: (i) 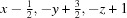; (ii) 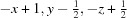; (iii) 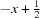, 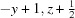.
