# Supplementary material for: HBr or not HBr? That is the question: crystal structure of 6-hy­droxy-1,4-diazepane-1,4-diium dibromide redetermined
Source: Acta Crystallogr C Struct Chem. 2019 May 16;75(Pt 6):678–85. doi: 10.1107/S2053229619005321 (PMC6550370; doi:10.1107/S2053229619005321)
Supplement: Supplementary file 3 [file c-75-00678-sup3.pdf]

Acta Cryst C

STRUCTURAL CHEMISTRY

Volume 75 (2019)

Supporting information for article:

HBr or not HBr? That is the question: crystal structure of 6-hydroxy-1,4-diazepane-1,4-diium dibromide redetermined

Mateusz Piontek, Bernd Morgenstern, Nils Steinbrück, Bastian Oberhausen, Guido Kickelbick and Kaspar Hegetschweiler

Full list of refcodes for CSD search

By studying some related literature, we became aware that the report of Liu *et al*. (1996) might not be the only example, where the nature of `Hbr' in a crystal structure should be questioned. A search in the latest version (2018, Version 5.20) of the Cambridge Structural Database (CSD; Groom *et al*., 2016), looking for molecular H---Br (*i.e.* for an H atom directly connected to a Br atom by a single bond) revealed a total of 69 entries:

ANOPEA, AWIVUY, BEPQIY, DAFFOH, EVAMIX, EWAZEJ, GICSOC, GIGYED, HACBOE, HEQGOZ, IVAWUY, JADVAM, KEGXUP, KEKQAS, KONVEO, KUFZOB, KUYQUS, MDOIMP, MIYZOK, MOKTAJ, MOMVIV, MOMVIV01, MPPYRL, MUFKON, MUSTEB, NAVFOI01, NIJGOC, NMTHFA01, PAJDIN, PASOHB, PEXHAB, PIMMAZ, PUPKOC, QEVSUF, QEVSUF01, QICGUF, QQQAQP, QQQASS, QQQATJ, QQQAVY, QQQGQY, SIJYAN, SOCZUH, TAZQAM, TMEPYR, TOKTIW (Liu's paper), TPPOBR, UFOYIX, UHEPII, UMTHFA01, UNESAI, VISBOP, VOKCII, YALFAS, YEBZEN, YISZUV, YITTOL,YOTSIJ, ZZZDIO, ZZZDNY, ZZZGUI, ZZZNGC, ZZZPOK, ZZZPSW, ZZZPTA, ZZZQRK, ZZZSZW, ZZZVJP, ZZZVLE.
